# Ionic Liquids and Deep Eutectic Solvents for Application in Pharmaceutics

**DOI:** 10.3390/pharmaceutics12100909

**Published:** 2020-09-23

**Authors:** Miguel M. Santos, Luís C. Branco

**Affiliations:** LAQV-REQUIMTE, Departamento de Química, Faculdade de Ciências e Tecnologia, Universidade Nova de Lisboa, 2829-516 Caparica, Portugal

Over the last few decades, Ionic Liquids (ILs) and Deep Eutectic Solvents (DES) have been studied academically throughout many fields of chemical and biological research, including pharmaceutical sciences, due to their highly tunable physical, chemical and physicochemical properties. In pharmaceutics, ILs have been studied as alternative solvents for the preparation, purification and crystallization of active pharmaceutical ingredients (APIs). However, the tailorable properties of ILs and DES render them distinct interactions with cellular membranes and organelles, enabling a myriad of applications ranging from bactericidal agents against pathological microorganisms to innovative materials for transdermal drug and protein delivery. ILs and DES have also found use in the manipulation of naturally occurring matrices such as polysaccharides, yielding outstanding materials suitable for tissue regeneration and wound healing, as well as gene and drug delivery. Furthermore, the formation of ILs by a combination of ionizable APIs with biocompatible organic counter-ions (API-ILs) has shown striking evidence over the last decade as a very capable instrument to enhance the bioavailability of poorly water and/or lipid-soluble drugs, in addition to reduce or even eliminate polymorphism, yielding more effective formulations of commercial drugs.

The goal of this Special Issue was to gather emergent discoveries on the application of Ionic Liquids and Deep Eutectic Solvents in pharmaceutical sciences. Six original papers and one review article were published.

The publication by Umerska & Tajber et al. [1] focused on anticrystal engineering of ketoprofen by combining it with benzocaine, procaine and tetracaine local anesthetics as Deep Eutectic Mixtures (DEMs). By melting and quench cooling a neat mechanical mixture of the components in discrete proportions, supercooled liquids were obtained for the vast majority of the prepared ketoprofen-based DEMs. A detailed thermal study was performed for all mixtures, which showed that the ketoprofen-procaine systems showed the most promising glass-forming ability of the three, with none of the components crystallizing when cooled or heated. In fact, this system was the only one that exhibited ionization, though partial, of ketoprofen’s carboxylic acid moiety by infrared spectroscopy and molecular modeling.

Three papers on the development of Organic Salts and Ionic Liquids from three different families of Active Pharmaceutical Ingredients (API-OSILs) with enhanced in vitro therapeutic activity were also included in this Special Issue [2,3,4]. On all papers, NMR and infrared spectroscopic studies as well as elemental analysis confirmed the formation of the highly bioavailable API-OSILs.

In more detail, Teixeira, Santos & Branco and co-workers [2] reported the combination of the anti-bone resorption drug alendronic acid (ALN) with four different cations in 1:1 and 2:1 proportions by deprotonation of one or two of the ALN phosphonate groups, respectively. In general, the monoanionic ALN-OSILs tended to be less toxic to healthy cells as well as to breast, lung and bone (osteosarcoma) cell lines than the dianionic ones. All ALN-OSILs displayed lower antitumor activity than the standard paclitaxel, however in several cases a distinction between healthy and cancer cells was observed. In this regard, the authors pointed towards [C_2_OHMIM][ALN] as the most promising ionic formulation of the set due to its efficiency towards lung cancer and osteosarcoma cell lines and very low toxicity towards fibroblasts.

By following one previously published methodology for the preparation of ampicillin-based OSILs [5], Ferraz & Branco and co-workers [3] reported the preparation of amoxicillin and penicillin antibiotics as OSILs bearing hydrolyzed β-lactam moieties (respectively *seco*-AMX- and *seco*-Pen-OSILs). Despite this shortcoming, on one hand, two of the hydrolyzed *seco*-Pen-OSILs showed up to a 100-fold increase in antimicrobial activity in vitro against sensitive *S. aureus* ([C_2_OHMIM][*seco*-Pen]) and *E. coli* ([TEA][*seco*-Pen]) strains compared to parent non-hydrolyzed antibiotics. The authors confirmed that the hydrolyzed version of the parent antibiotics, as Na or K salts, did not affect these bacteria; hence, there must be a distinct underlying mechanism for the antimicrobial activity of these *seco*-Pen-OSILs. On the other hand, the combination of both hydrolyzed antibiotics with [Choline], [P_6,6,6,14_] and [C_16_Py] cations efficiently inhibited the growth of resistant *E. coli* and MRSA strains in vitro. In fact, [C_16_Py][*seco*-AMX] recorded an increase of at least 1000-fold in comparison with the original antibiotic bearing an intact or hydrolyzed β-lactam moiety.

Santos & Branco and co-workers [4] reported the preparation of twelve new ciprofloxacin- and norfloxacin-based OSILs by combination with six different organic cations. The obtained isomorphous salts and ionic liquids were found to be highly water-soluble as well as non-toxic to mouse fibroblasts. The in vitro antimicrobial activity studies performed on sensitive pathogenic *K. pneumoniae* and *S. aureus* strains, as well as commensal *B. subtilis*, revealed that it is possible to develop fluoroquinolone-based OSILs with very distinct activities, depending on the suitable combinations with organic cations. For example, the combination of [C_16_Py] cation with ciprofloxacin and norfloxacin selectively inhibited the growth of *K. pneumoniae* (20-fold) and *S. aureus* (11-fold), respectively, with a reduced effect on the other strains. On the other hand, the combinations of ciprofloxacin with [EMIM] or [C_2_OHDMIM] cations are also promising, as they have enhanced activity against both pathogenic bacteria strains.

Two additional research papers on the development of lipid-based formulations (LBFs) of water-insoluble drugs by means of Ionic Liquids were also included in this Special Issue. The report by Tay & Porter et al. [6] focused on the dissolution profile of lumefantrine combined with docusate and dodecyl sulfate in nine different LBFs. Upon spectroscopic and physicochemical characterization of the two ILs, solubility studies showed particularly promising loadings with the docusate IL in four of the LBFs. Type III formulations were able to maintain solubilization and supersaturation of the drugs in vitro, which consequently prompted a 35-fold plasma exposure in vivo than with the remaining tested formulations. Moreover, in comparison with lipid and aqueous suspensions of the drug’s free base, this formulation achieved respectively 10 and 50 times more oral bioavailability.

On the other hand, Islam & Goto et al. [7] prepared promising Ionic Liquid-in-oil microemulsions (IL/O ME) for the transdermal delivery of acyclovir. The MEs were composed of hydrophilic ILs, which were based on the combination of choline with formate, lactate and propionate, and choline oleate as the surfactant IL in combination with Span-20. The highly stable nano-sized droplets of ME allowed up to 7.7 mg/mL drug loadings (with the propionate salt) and significantly enhanced drug permeation across pig skin models by reduction of the skin barrier function with very high biocompatibility.

Transdermal drug delivery systems assisted by Ionic Liquids were also the focus of the review article by Sidat & Pillay et al. [8]. The physical, chemical and physicochemical properties of the ILs are discussed according to their lipophilic and hydrophilic behavior, as well as their possible interactions with biomolecules and biological membranes. The authors point out several examples in which ILs are used as skin permeation enhancers in both seldom or synergistic combinatorial use with striking advantages over standard systems. Moreover, the dissolution and complexation of ILs with drugs, as well as the preparation of ILs as Active Pharmaceutical Ingredients for assembly of transdermal drug delivery systems, have been revised.
